# The views of parents of children with cancer and pediatric physical therapists on a network for continuity and optimal quality of care for children with cancer: KinderOncoNet

**DOI:** 10.1007/s00520-023-08211-6

**Published:** 2023-12-06

**Authors:** L. B. Kleinlugtenbelt, W. J. E. Tissing, W. J. M. Plieger-van Solkema, P. van der Torre, W. J. W. Kollen, J. W. Gorter

**Affiliations:** 1 Princess Máxima Center for Pediatric Oncology, Heidelberglaan 25, 3584 CS Utrecht, The Netherlands; 2 Department of pediatric oncology, University of Groningen, University Medical Center Groningen, Groningen, The Netherlands; 3 Dutch Childhood Cancer Organisation, Utrecht, The Netherlands; 4 Department of Rehabilitation, Physical Therapy Science and Sports, University Medical Center Utrecht, Utrecht, The Netherlands; 5 UMC Utrecht Brain Center and Center of Excellence for Rehabilitation Medicine, Utrecht University, Utrecht, The Netherlands

**Keywords:** Childhood oncology, Care network, Pediatric physiotherapy, Quality of life, Supportive care

## Abstract

**Purpose:**

Children with cancer require specific therapeutic guidance. Parents prefer physical therapy close to home, while pediatric physical therapists (PPTs) working in the community may lack specific knowledge. The aim of this study is to determine the needs of parents of children with cancer and PPTs to inform the design and development of a care network, named “KinderOncoNet.”

**Methods:**

We explored the perspectives and needs of parents of children with cancer and PPTs in the community, and we investigated the added value that KinderOncoNet could offer. We used an iterative process; data collection consisted of (1) gathering information from parents of children with cancer and PPTs through a survey and (2) co-creation sessions with stakeholders.

**Results:**

In total, 98 parents and 177 PPTs participated in the survey. Parents (97%) and PPTs (93%) indicated that the care network would bring added value. All but one parent stressed the importance of a local PPT being aware of both the condition and the side and late effects of oncological treatment. Moreover, 40% of PPTs thought they do not have sufficient knowledge to provide high-quality therapy and that they would embrace opportunities for education. Through the co-creation sessions, a prototype of the care network was conceptualized.

**Conclusion:**

KinderOncoNet can contribute to the continuity and quality of physiotherapy care for children with cancer during and after the oncological treatment. Such a network would allow for sharing knowledge, developing skills, and improving accessibility and communication in the Netherlands.

## Introduction

In the Netherlands, around 600 children are diagnosed with cancer each year [1]. The treatment of childhood cancer often comes with many side effects. The increasing survival due to more intensive treatment protocols has come with an increased number of side effects. With higher cure rates, the total number of childhood cancer survivors increases, resulting in increasing numbers of patients with short- and long-term medical, physical, and psychosocial needs, often extending into adulthood [2, 3]. As a consequence of more side effects and increasing numbers of survivors, [4] pediatric physiotherapists (PPTs) are seeing more children and adolescents with cancer, during and after oncological treatment.

Since 2018, the care for children and adolescents with cancer in the Netherlands is centralized at the Princess Máxima Center in Utrecht. The mission is to cure every child with cancer with optimal quality of life. Our center aims to centralize care when needed and to provide care locally when possible. Parts of the oncological treatment can be administered in 1 of 15 shared care centers. Our hypothesis is that this care model could be further optimized by extending the continuity of physiotherapy care to close to home and community settings and by sharing our knowledge, expertise, and experience with professionals.

Currently, pediatric physiotherapy for children with cancer is delivered by PPTs in the Sports and Exercise Center of the Princess Máxima Center while children are admitted or undergo medical treatment or by the local pediatric physiotherapist when the patients are at home. In 2021, 304 of the 553 (55%) newly diagnosed children were seen by a pediatric physiotherapist in the Princess Máxima Center, with 225 of the 553 (41%) of these children being referred to a local PPT after discharge.

The physiotherapy program in the Princess Máxima Center is tailored to the needs of each child, adolescent, and family, depending on the type of cancer, developmental stage (0–18 years), phase of the oncological trajectory (before, during, or after treatment), and any complications and/or late effects of the treatment. Pediatric physical therapy can be helpful both to reduce the impact of side effects of the oncological treatment with respect to impairment level (such as balance impairments due to polyneuropathy) and to improve activity (e.g., walking) and participation (e.g., school, sports), and overall quality of life of the children [5, 6]. The premise is that when children with cancer are better equipped and supported across settings, it will lead to better physical and mental health outcomes, as well as better functioning in society, and to less costs in the future [7–10].

While continuity of care by a PTT at home is critically important to reduce side and late effects of the oncological disease [11, 12], we currently lack a network of specialized PPTs in cancer across settings in the Netherlands. Most of the local PPTs in the Netherlands only see none to four children with childhood oncology in their whole career. With all the different types of cancer and various needs, a PPT in the community will thus always lack knowledge and experience. Therefore, to optimize continuity and quality of care by physiotherapists close to home, it makes sense that PPTs need access to up-to-date knowledge and expertise related to pediatric oncology. Developing a care network could be a solution to overcome this care gap.

Worldwide, there is an understanding that the concept of network medicine is critical to meet the needs of the growing population of childhood cancer survivors [13]. Facilitating the right care in the right place is one of the most important objectives of care networks. This approach means not only prevention of more expensive care in specialized centers, but also to move care closer to people’s homes, whenever possible, for better quality and efficiency [14–16]. Goals of such healthcare networks often have a quadruple aim, i.e., improving the patient experience of care, the health of populations, reducing per capita costs, and improving the work life of those who deliver care [17]. Looking at existing care networks and best practices in the Netherlands, such as CP-NET (cerebral palsy), OncoNET (oncology for adults), CVA-Net (stroke), and ParkinsonNet (Parkinson disease) [18], or internationally like ActiveOncoKids [19, 20], the National Physical Activity and Childhood Cancer Network in Australia [21], the Italian rehabilitation group [22], and Pogo [23], we know that network care can improve the quality of care. In line with these existing networks, we aim to improve the pediatric physiotherapy care across settings for children by establishing a care network, KinderOncoNet (Children’s Oncology Network).

The primary objective of this study is to determine the needs of parents of children with cancer and PPTs across all settings, to inform the design and development of a care network. In addition, we asked stakeholders about the added value that KinderOncoNet could offer.

## Method

### Study setting and design

We initiated a project named “KinderOncoNet” in the Princess Máxima Center for pediatric oncology in Utrecht, the Netherlands, from March 2021 to March 2022. The study was designed as an iterative process, to determine the needs and added value for our care network. The project group included the following members and stakeholders (total *n* = 8): two professionals from the Princess Maxima Center (a PPT/project leader and a PPT/manager of the department Sports and Exercise); an employee of the parent/patient organization: the Dutch Childhood Cancer Organisation (VKKN); a board member of the Dutch Association for Pediatric Physical Therapy (NVFK); an employee of Enter Communication (an ICT company); an employee from the HU (University of Applied Sciences) Utrecht, the Netherlands; lectorate innovations and exercise care; and two students from the Bachelor of Physical Therapy Program at the HU in Utrecht, the Netherlands. Data collection consisted of two different phases: gathering information from the parents of children with cancer, during and after treatment and from PPTs through a survey (phase 1), and co-creation sessions with stakeholders, to further expand on the knowledge obtained in phase 1 (phase 2).

### Ethical approval

All procedures involving human participants were in accordance with the ethical standards of the institutional and/or national research committee and with the 1964 Helsinki Declaration and its later amendments or comparable ethical standards. The study, a quality improvement project, is not considered subject to the Medical Research Involving Human Subjects Act (WMO) [24]. This study does not fall under the scope of the Dutch Medical Research Involving Human Subjects Act (WMO). It therefore does not require approval from an accredited medical ethics committee in the Netherlands.

### Selection method

The survey for parents was distributed online by the Princess Máxima Center’s newsletter for parents, the Dutch Childhood Cancer Organisation, and a Facebook group for parents. Participation was voluntary. The survey for PPTs was distributed online by the the Dutch Society for Pediatric Physiotherapy (NVFK) to affiliated PPTs in the Netherlands across all settings in the community. The survey asked participants to indicate whether they were interested in joining the consortium. The consortium partners were purposefully selected by the project group to ensure representative PPTs across all settings (primary care, shared care center, and rehabilitation center).

### Data collection

#### Phase 1: survey

Needs of parents of children with cancer during and after treatment, and PPTs, and also beliefs about the added value to develop a knowledge platform and care network for establishing KinderOncoNet were determined by a survey. The survey consisted of closed and open-ended questions and statements with Likert scale (0, not important, to 10, very important). The survey for parents consisted of ten questions (closed and open-ended) about (1) parents’ experiences with physical therapy care in childhood cancer and (2) parents’ need for developing a care network. The survey for PTTs consisted of 36 questions about (1) current workplace, (2) experience with and knowledge of pediatric oncology, and (3) expectations of a pediatric oncology exercise care network and knowledge platform. Open-ended questions were used to explore their expectations in developing a network.

#### Phase 2: co-creation sessions

Based on the results of the surveys, two co-creation sessions were organized with consortium partners and stakeholders (see the “Method” section) to further explore what the needs for PPTs are and to convert this in ideas to create a network, based on an action-oriented research approach [25]. In co-creation sessions, good and productive collaboration among every participant is very important and is taken into consideration from the very early steps in conceptualizing the prototype [26, 27].

The first co-creation meeting was organized in May 2021. This meeting took place online by Zoom because of the restrictive measures regarding COVID-19. The second co-creation meeting was organized in September 2021. This meeting took place in-person at the Princess Máxima Center in Utrecht, the Netherlands. The theme of the first’s co-creation meeting was “find, connect, and trust” and aimed to identify the needs in terms of collaboration and findability within the future care network, KinderOncoNet. Topics of accessibility, privacy, and communication capabilities were also discussed.

The theme of the second in-person co-creation meeting was “knowledge and expertise.” Based on the questions of the survey, topics as “what is needed to improve capacity and competence?,” “how to maintain knowledge and expertise?,” and “which educational needs and what content and form of educational needs within the care network are necessary?” were further explored and discussed. Also, the conditions to participate in KinderOncoNet were determined.

### Statistics

Data of the survey (phase 1) were collected by SurveyMonkey, and descriptive statistics (frequency, distribution) were generated by Excel [28].

Data from the co-creation meetings were collected by note taking during the sessions and analyzed and coded through a tree structure of the topics using the software ATLAS.ti [29].

## Results

### Stakeholder engagement

We used a multi-stakeholder approach of parents and PPTs in phase 1 (survey) and PPTs across all settings (primary care, shared care center, and rehabilitation center) in phase 2 (co-creation sessions) see Table 1.

**Table 1:** Participants in the survey and co-creation sessions

Participants in survey		Total (*N*)	Range (mean; sd)
Parents		98	
During treatment		39	
After treatment		59	
Treatment of a local PTT during treatment?	*Yes*	79	
	*Yes, but not local*	8	
	*No*	12	
PPTs		177	
Gender	*Female*	158	
	*Male*	15	
Age (years)			22–70 (42.4; 10.7)
Work experience (years)			0–44 (16.3; 10.2)
Setting	*Primary care*	154	
	*Secondary care (incl. shared care and rehabilitation center)*	24	
	*Tertiary care*	5	
Participants in co-creation sessions			
PTTs in the community*		34	
PPTs** from primary care settings		19	
PPTs from shared care center		6	
PTTs nine from rehabilitation center		9	

*Actively involved consortium of PPTs nationwide with knowledge and expertise to carry out the research. The participants were recruited by a call from the NVFK. To select participants for the consortium group, conditions were set for representing primary, secondary, and tertiary care and shared care center across the Netherlands

**PPTs pediatric physiotherapists

#### Phase 1: survey

In total, 98 parents and 177 PPTs participated in the survey. For characteristics of participants of the survey, see Table 1.

In total, 267 out of 275 (97%) participants (parents and PPTs) expressed added value in the development of a knowledge platform and care network. In total, 94 out of 98 (96%) of the parents indicated that it is important that the local PPT is aware of not only the pediatric oncological condition but also the side effects and late effects of oncological treatment affecting exercise (mean 9.0 on a scale of 1–10). Parents expected to be referred from the Princess Máxima Center to a local PTT with expertise and experience in pediatric oncology (7.7 on a scale of 0–10). Eighty percent of the parents are willing to travel 15–30 min from home to a local PPT. For further outcomes, see Table 2.

**Table 2:** Summary of outcome survey parents (*n* = 98)

Question	Answered	Outcome
Experience with pediatric physiotherapy	96	Range (0–10); mean 7.5
When I come to the Princess Máxima Center, I expect to be referred by the Maxima to a pediatric physiotherapist with expertise in pediatric oncology	95	Range (0–10); mean 7.7
I find it important that the pediatric physiotherapist has experience and up-to-date knowledge in children with an oncological condition	98	Range (0–10); mean 9.0
For a pediatric physical therapist with pediatric oncology knowledge and experience, I am willing to invest the following additional travel time	98	No extra travel time; 11 (11.2%) 15 min; 31 (31.6%) 30 min; 47 (48.0%) Max 60 min; 16 (16.3%)
KinderOncoNet has to be assessable for parents?	98	Yes; 87 No; 11

Results from the questionnaire for PPTs are presented in Table 3. Forty percent of the PPTs mentioned they do not have sufficient knowledge to be able to provide a high-quality therapy (on a scale of poor, insufficient, sufficient, more than sufficient, excellent, see Table 3). PPts mentioned the lack of opportunities for education to gain more knowledge in the field of pediatric oncology and physiotherapy care. Thirty percent of the PPTs indicated that they needed education to become more competent in treating a child with the diagnose childhood cancer, and fifteen percent of the PPTs indicated that experience is important to become more competent in the field of pediatric oncology. Also, an up-to-date knowledge platform (8.3%), intervision and peer review in the professional field (6.3%), and case management (4.1%) were mentioned to be important. Other topics PPTs provided in response to the final open-ended question asking about suggestions for the knowledge hub were (1) knowledge about specific exercise physiology and treatment in children with childhood cancer (36.5%), (2) pediatric oncology in general (30.4%), (3) red flags (11.5%), and (4) psychological counseling (7.4%). In addition, the effects of chemotherapy on the child (6.1%), side effects of medication (5.4%), and information about different prognoses (3.4%) were mentioned to a lesser extent.

**Table 3:** Summary of outcome survey PPTs (*n* = 177)

Question	Answered (*n*)	Outcome	Percentage
How many children with cancer in treatment during career?	175	0 (*n* = 15) 1–4 (*n* = 78) 5–10 (*n* = 57) 11–20 (*n* = 14) > 20 (*n* = 11)	8.6% 44.6% 32.6% 8.0% 6.3%
Willing to connect to KinderOncoNet?	163	Yes (*n* = 153) No (*n* = 10)	93.9% 6.1%
KinderOncoNet is of added value?	164	Yes (*n* = 153) No (*n* = 11)	93.3% 6.7%
KinderOncoNet accessible for parents?	168	Yes (*n* = 107) No (*n* = 54)	66.5% 33.5%
General knowledge childhood oncology	168	Poor (*n* = 4) Insufficient (*n* = 61) Sufficient (*n* = 87) More than sufficient (*n* = 14) Excellent (*n* = 2)	2.4% 36.3% 51.8% 8.3% 1.2%
General knowledge hemato-oncology	167	Poor (*n* = 11) Insufficient (*n* = 69) Sufficient (*n* = 69) More than sufficient (*n* = 17) Excellent (*n* = 1)	6.6% 41.3% 41.3% 10.2% 0.6%
General knowledge	168	Poor(*n* = 13) Insufficient (*n* = 72) Sufficient (*n* = 68) More than sufficient (*n* = 12) Excellent (*n* = 3)	7.7% 42.9% 40.5% 7.1% 1.8%
Solid tumors	164	Poor (*n* = 13) Insufficient (*n* = 85) Sufficient (*n* = 55) More than sufficient (*n* = 9) Excellent (*n* = 2)	7.9% 51.8% 33.5% 5.5% 1.2%
General knowledge bone tumors	167	Poor (*n* = 13) Insufficient (*n* = 77) Sufficient (*n* = 60) More than sufficient (*n* = 14) Excellent (*n* = 3)	7.8% 46.1% 35.9% 8.4% 1.8%
General knowledge neuro-oncology	168	Poor (*n* = 13) Insufficient (*n* = 72) Sufficient (*n* = 68) More than sufficient (*n* = 12) Excellent (*n* = 3)	7.7% 42.8% 40.5% 7.1% 1.8%
Sufficient knowledge after Master Pediatric Physical Therapy	165	Poor (*n* = 13) Insufficient (*n* = 66) Neutral (*n* = 67) Sufficient (*n* = 18) More than sufficient (*n* = 1)	7.9% 40.0% 40.6% 10.9% 0.6%

Furthermore, availability of online e-learnings, physical education, and organization of network meetings were mentioned. Regarding the accessibility of KinderOncoNet, the establishment of an active forum for easy contact with colleagues, acceptable costs, and a not too high investment of time to be part of the network were mentioned. Moreover, participation in the network should be reimbursed by health insurance companies, and CME points should be granted for the education.

#### Phase 2: co-creation sessions

The co-creation sessions were attended by *n* = 31 (session 1) and *n* = 25 (session 2) PPTs of the in total 34 stakeholders in the project (*n* = 19 pediatric physiotherapists from primary care settings, *n* = 6 from shared care center, and *n* = 9 from rehabilitation center) (see Table 1).

The theme of the first’s co-creation meeting was “find, connect, and trust” and aimed to identify the needs in terms of collaboration and findability within the future care network, KinderOncoNet. Topics of accessibility, privacy, and communication capabilities were also discussed. In the first co-creation session, it was indicated that PPTs outside the Maxima need good access to professionals within the Princess Maxima Center for consultation and patient discussions. Moreover an easy and secure way to share confidential information and files would facilitate collaboration across settings.

Secondly, trust is very important. Thresholds to treat a child with childhood cancer should be removed. It is therefore important that referrals are made to each other with additional information, knowledge, and clear indications and that people know the limits of their own competences and knowledge.

The expectations of KinderOncoNet lay mainly in the creation of an accessible network in which it is easy to communicate, where colleagues can easily be found and where up-to-date knowledge and training are offered. Connection to KinderOncoNet should not take too much time, be affordable, and should have a form that ensures an active connection.

The theme of the second in-person co-creation meeting was “knowledge and expertise.” Based on the questions of the survey, the following topics were further explored and discussed: “what is needed to improve capacity and competence?,” “how to maintain knowledge and expertise?,” and “which educational needs and what content and form of educational needs within the care network are necessary?” Also, the conditions to participate in KinderOncoNet were determined. During the second co-creation meeting, the referral from care from the Princess Maxima Center to care close to home, so the transfer between different institutions, was mentioned to be a very important subject. All children diagnosed with childhood cancer start their treatment in the Princess Maxima Center, and all complex care takes place in Utrecht. Less intensive care is provided in the shared care centers closer to home. As diagnosis is established in the Princess Máxima Center, and treatment is started there; the transfer to another institution for further treatment is difficult. The idea from the participants was to initiate this familiarization and transfer early in the treatment process, so that child and caregivers are already familiar with professionals closer to home. So finding a PPT close to home from the beginning of the therapy would be helpful. Appointing a case manager could improve the transition and communication. Thereby, it was found to be important that different institutions can reinforce and complement each other in sharing care, to get the right care in the right place.

About education and gaining knowledge, it was often mentioned that both physical and online trainings are desirable. The content of these trainings can cover general knowledge about pediatric oncology, related treatments, fatigue symptoms, and cognitive, traumatic, and psychosocial support. In addition, it was mentioned that annual trainings with the possibility of deepening through e-learnings is needed. During the physical training, there should also an opportunity to find each other, to connect, and to network with each other; attendance at physical days may be mandatory to encourage active membership. Finally, the care network and knowledge platform should be accessible to parents, children, and survivors, so they can find a competent professional close to home and be expanded to other allied healthcare professionals (dieticians, occupational therapists, and speech and language therapists) and psychosocial disciplines. The result of this study is a prototype of the knowledge platform KinderOncoNet based on the needs of the participants in this study.

## Discussion

In this study, we determined that almost all parents indicated a need for a local PPT who is knowledgeable about both the pediatric oncological condition, its side effects, and late effects of oncological treatment affecting exercise. At the same time, almost half of the PPTs indicated a gap in knowledge to be able to provide high-quality therapy and also a need for education in the field of pediatric oncology and physiotherapy care. There was a general understanding that the development of a care network for pediatric oncology would improve care, KinderOncoNet that facilitates collaboration, communication, and trust in each other.

The findings provide support for the idea to create a care network, which is in line with existing initiatives and research. Firstly, we know from care networks such as Parkinson disease (ParkinsonNet) [18] in the Netherlands and international initiatives for childhood cancer, like ActiveOncoKids [19, 20]; the National Physical Activity and Childhood Cancer Network in Australia [21]; the Italian rehabilitation group [22]; and Pogo [23] that network care can improve the quality of care [14]. Moreover, our initiative would be in line with the directions provided by the Ministry of Health, Welfare and Sport in the Netherlands. In the report of their taskforce “The Right Care in the Right Place,” network care is the answer to the existing friction between a life-transcending demand for care for (chronic) conditions and the care landscape clustered in disciplines and care lines [30]. KinderOncoNet could be the solution for the challenge with the small number of children diagnose with cancer in need of optimal quality and continuity of care, the right care at the right place.

Secondly, KinderOncoNet might improve knowledge. Results from a study from Gohar [31] show that although physicians identified musculoskeletal complications in children with cancer, only a minority of these patients were referred for PT. This knowledge supports the need for increasing the awareness of physicians about benefits of early integration of PT into the therapy plan for a child and to refer them as soon as possible to a PPT close to home [31]. Moreover, there is evidence in the importance of PPT in reducing physical function problems during and after treatment to keep them as healthy as possible [6, 11, 12, 32]. So when children get more optimal referred to a PPT close to home, through the collaboration within KinderOncoNet, the quality and continuity of care in physical function improves. Of note, the number of childhood cancer survivors is increasing. In 2020, the number of childhood cancer survivors (CCS) in Europee reached 500,000 [33, 34], of which many experience late effects [35, 36]. Most young adults reported a need for support, in particular for information, especially regarding lifestyle and health risks after childhood cancer. It is important to empower them to take control over their health [32]. Thus, thirdly, KinderOncoNet has the potential to be the hub, connecting patients, parents, survivors, and healthcare professionals in the Princess Máxima Center with professionals closer to home and facilitate the easy access to knowledge and skills about these rare diseases. By doing so, KinderOncoNet would indeed be the solution to find the right healthcare professional close to home.

### Strengths and limitations

Strengths of our study are that the iterative process in two phases allowed us to first analyze the data from the survey of a large number of participants and then categorize the information into themes as input for the smaller co-creation sessions.

Another strength is that the project study group included representation of the VKKN and a large number of parents of children with cancer. The literature demonstrates that engagement of stakeholders has great benefits, through partnership in research, patients, and healthcare professionals are actively involved throughout the entire research process [37–40]. The use of co-creation meetings brought the study closer to the stakeholders and ensured a higher potential for impact.

However, some limitations of the study must be acknowledged. This study focused only on physical therapy. However, there is also a wide range of other healthcare disciplines involved in the care of a child with cancer. A broader scope of KinderOncoNet could help improve its goal. Therefore, we plan to expand KinderOncoNet for other healthcare professionals, including dieticians, speech and language therapists, occupational therapists, and psychologists. We should also expand the target population to those children who are receiving long-term follow-up care, so-called childhood cancer survivors, and those transitioning from pediatric care into adult healthcare settings. Another limitation is the recruitment of participants. The recruitment was voluntary, for the parents and PPTs. This may have created a stakeholder group consisting mainly of partners with a high sense of participation [41]. Based on the results of this study, we described expectations, wishes, and products to conceptualize and develop KinderOncoNet (see Table 4). In the future, the prototype will be developed and evaluated in clinical practice and among other disciplines, such as psychology, speech therapy, occupational therapy, and dietetics. Once KinderOncoNet is sufficiently functional and deployable, further research will be started to show its feasibility and effectiveness, on the continuity and quality of care for children with childhood oncology during and after treatment. We envision a future evaluation of the costs and benefits of the national network using the quadruple aim framework to report on the impact on population health, healthcare experiences, costs, and professional experiences.

**Table 4:** Expectations, wishes, and products to conceptualize and develop KinderOncoNet

Recommendations	
• Knowledge platform, with up-to-date information for child/parents, survivors, and allied healthcare professionals	
• Realization of a multidisciplinary digital care access map for parents, children, and healthcare professionals, where they can find each other, and it is visible which quality requirements they meet and to find the nearest allied healthcare professional close to home	
• Development of a multidisciplinary “professional in pediatric oncology” training and e-learnings for allied healthcare professionals	
• Define responsibilities and care processes agreements within the care network of allied health professionals	
• Connect for optimal quality and continuity of care close to home!	

## Conclusion

Parents of children with cancer and PPTs clearly indicate that there is a need to develop a national care network specialized in pediatric oncology. Through this project, we now have a conceptual network “KinderOncoNet” that will facilitate involvement of children/parents/survivors and PPTs across settings to improve the accessibility, continuity and quality of care, participation, and quality of life in the Netherlands.

## Data Availability

The data that support the findings of this study are available on request from the corresponding author.

## References

[1] SKION "http://www.skion.nl". Accessed 30 Nov 2023

[2] Verdecchia A (2007). Recent cancer survival in Europe: a 2000-02 period analysis of EUROCARE-4 data. Lancet Oncol.

[3] Landier W (2018). Surveillance for late effects in childhood cancer survivors. J Clin Oncol.

[4] Schulpen M (2021). Significant improvement in survival of advanced stage childhood and young adolescent cancer in the Netherlands since the 1990s. Eur J Cancer.

[5] Gaser D (2022). Effects of strength exercise interventions on activities of daily living, motor performance, and physical activity in children and adolescents with leukemia or non-Hodgkin lymphoma: results from the randomized controlled ActiveADL study. Front Pediatr.

[6] Braam KI (2016). Physical exercise training interventions for children and young adults during and after treatment for childhood cancer. Cochrane Database Syst Rev.

[7] Wurz A (2019). Physical activity programs for children diagnosed with cancer: an international environmental scan. Support Care Cancer.

[8] Scott JM (2018). Association of exercise with mortality in adult survivors of childhood cancer. JAMA Oncol.

[9] Gotte M, Taraks S, Boos J (2014). Sports in pediatric oncology: the role(s) of physical activity for children with cancer. J Pediatr Hematol Oncol.

[10] Götte M (2016). Translation of exercise research into practice models in children and adolescents with cancer. Oncol Res Treat.

[11] Morales JS (2020). What are the effects of exercise training in childhood cancer survivors? A systematic review. Cancer Metastasis Rev.

[12] Stossel S (2020). Benefits of exercise training for children and adolescents undergoing cancer treatment: results from the randomized controlled MUCKI trial. Front Pediatr.

[13] Howell D (2012). Models of care for post-treatment follow-up of adult cancer survivors: a systematic review and quality appraisal of the evidence. J Cancer Surviv.

[14] Parkinsonnet, parkinsonnet__het_wetenschappelijk_bewijs_meta.pdf. https://www.parkinsonnet.nl/app/uploads/2019/11/parkinsonnet__het_wetenschappelijk_bewijs_meta.pdf. Accessed 30 Nov 2023

[15] Integraal Zorgakkoord: 'Samen werken aan gezonde zorg' | Rapport | Rijksoverheid.nl

[16] 2025, D.M.S., https://www.demedischspecialist.nl/onderwerp/medisch-specialist-2025. Accessed 30 Nov 2023

[17] Bodenheimer T, Sinsky C (2014). From triple to quadruple aim: care of the patient requires care of the provider. Ann Fam Med.

[18] Munneke M (2010). Efficacy of community-based physiotherapy networks for patients with Parkinson’s disease: a cluster-randomised trial. Lancet Neurol.

[19] Gotte M (2022). Multidisciplinary Network ActiveOncoKids guidelines for providing movement and exercise in pediatric oncology: consensus-based recommendations. Pediatr Blood Cancer.

[20] https://www.activeoncokids.org/. Accessed 30 Nov 2023

[21] https://littlebigsteps.org.au/. Accessed 30 Nov 2023

[22] Rossi F (2019). A new frontier of pediatric oncology rehabilitation: establishment of a dedicated working-group within the Italian Association of Pediatric Hematology and Oncology. J Pediatr Rehabil Med.

[23] https://www.pogo.ca/. Accessed 30 Nov 2023

[24] https://english.ccmo.nl/investigators/legal-framework-for-medical-scientific-research/your-research-is-it-subject-to-the-wmo-or-not. CCMO. Accessed 30 Nov 2023

[25] Driessen-Willems MD, Bartelink NHM, Bessems KMHH, Kremers SPJ, Kintzen C, van Assema P (2021) Co-creation approach with action-oriented research methods to strengthen “Krachtvoer”; a school-based programme to enhance healthy nutrition in adolescents. Int J Environ Res Public Health 18(15):7866. 10.3390/ijerph18157866 10.3390/ijerph18157866 PMC8345603 34360158

[26] Galvagno M, Dalli D (2014). Theory of value co-creation: a systematic literature review. Manag Serv Qual.

[27] Saha V, Goyal P, Jebarajakirthy C (2022). Value co-creation: a review of literature and future research agenda. J Bus Ind Mark.

[28] Waclawski E (2012). How I use it: Survey Monkey. Occup Med (Lond).

[29] Soratto J, Pires DEP, Friese S (2020). Thematic content analysis using ATLAS.ti software: potentialities for researchs in health. Rev Bras Enferm.

[30] https://www.tweedekamer.nl/kamerstukken/detail?id=2019D26435&did=2019D26435

[31] Gohar SF, Marchese V, Comito M (2010). Physician referral frequency for physical therapy in children with acute lymphoblastic leukemia. Pediatr Hematol Oncol.

[32] van Erp LME (2022). Support needs of Dutch young adult childhood cancer survivors. Support Care Cancer.

[33] Vassal G, Ladenstein R, Schrappe M, Pritchard-Jones K, Biondi A (2016). The SIOPE strategic plan: a European cancer plan for children and adolescents. J Cancer Policy.

[34] Chilhood Cancer International (CCI) “It’s not over when it’s over” Available from: https://ccieurope.eu/#:~:text=Currently%2C%20there%20are%20500%2C000%2B%20childhood%20cancer%20survivors%20in%20Europe, 2021. Accessed 30 Nov 2023

[35] Oeffinger KC (2006). Chronic health conditions in adult survivors of childhood cancer. N Engl J Med.

[36] Bhakta N (2019). Childhood cancer burden: a review of global estimates. Lancet Oncol.

[37] Pozniak K (2021). Building a culture of engagement at a research centre for childhood disability. Res Involv Engagem.

[38] Bult-Mulder M, Vd KJ, Voorman J, Willems-ophet Veld M, Gorter JW, en Ketelaar M (2023). Duurzame samenwerking met ervaringsdeskundigen in onderzoek en innovatie; wat, hoe en hoe verder?. Nederlands Tijdschrift voor Revalidatiegeneeskunde.

[39] Ketelaar M, Smits DW, van Meeteren K, Klem M, Alsem M, Imms C, Green D (2020). Involvement of young people and families in all stages of research: what, why and how?. Participation: optimising outcomes in childhood-onset neurodisability.

[40] Smits DW (2020). Designing a tool to support patient and public involvement in research projects: the Involvement Matrix. Res Involv Engagem.

[41] Grol R, W.M., redactie. Implementatie. 4de druk. Effectieve verbeteringen van patiëntenzorg. Amsterdam: Reed Business; 2011. Docentenrol

[42] https://regieorgaan-sia.nl/financiering/raak-publiek/. Accessed 30 Nov 2023

[43] https://projecten.zonmw.nl/nl/project/development-and-evaluation-national-network-allied-health-professionals-working-children. Accessed 30 Nov 2023

